# Ectopic Thyroid Mass Separately Present in Mediastinum and Not a Retrosternal Extension: A Report of Two Cases

**DOI:** 10.1155/2019/3821767

**Published:** 2019-03-12

**Authors:** Abdul Ahad Sohail, Syed Shahabuddin, Moghira Iqbaluddin Siddiqui

**Affiliations:** ^1^The Aga Khan University Hospital, Karachi, Pakistan; ^2^Department of Surgery, The Aga Khan University Hospital, Karachi, Pakistan

## Abstract

Retrosternal extension of goiter is one of the most common types of masses in the superior mediastinum. These types of goiters classically present with compressive symptoms such as dyspnea, dysphonia, dysphagia, or sleep apnea. Surgical treatment with a total thyroidectomy and complete removal of the intrathoracic portion of thyroid is the gold standard treatment. These cervicomediastinal lesions at times may not be continuous, and a sternotomy may be required for complete and safe excision of the mediastinal mass to achieve decompression of the surrounding structures and preventing the hemorrhagic complications if attempted from cervical incision. We present a summary of two cases that gave an initial impression of retrosternal extension of thyroid gland, however intraoperatively were found to be separately encapsulated and required sternotomy for its safe and complete excision.

## 1. Introduction

Retrosternal extension of goiter is one of the most common types of masses in the superior mediastinum [1, 2]. Although a clear definition is not present in the literature for retrosternal, substernal, or mediastinal goiter, it usually denotes the extension of thyroid tissue from its cervical portion in continuity passing into the anterior mediastinum, anterior to the arch of aorta [2]. These type of goiters classically present with compressive symptoms such as dyspnea, dysphonia, dysphagia, or sleep apnea, and less commonly, these masses can compress the neurovascular structures resulting in superior vena cava syndrome and Horner's syndrome [3, 4]. Therefore, surgical treatment with a total thyroidectomy and complete removal of the intrathoracic portion of thyroid is the gold standard treatment via a cervical approach or in rare circumstances a partial or complete median sternotomy may be required for complete and safe excision of the mediastinal mass achieving the decompression of the surrounding structures and preventing the hemorrhagic complications [5, 6]. One of the interesting features of these cervicomediastinal lesions is that they may not be continuous. We report two cases of cervicomediastinal goiter expected to be in continuity but intraoperatively observed to be close but separately capsulated.

## 2. Case 1

**Figure 1:**
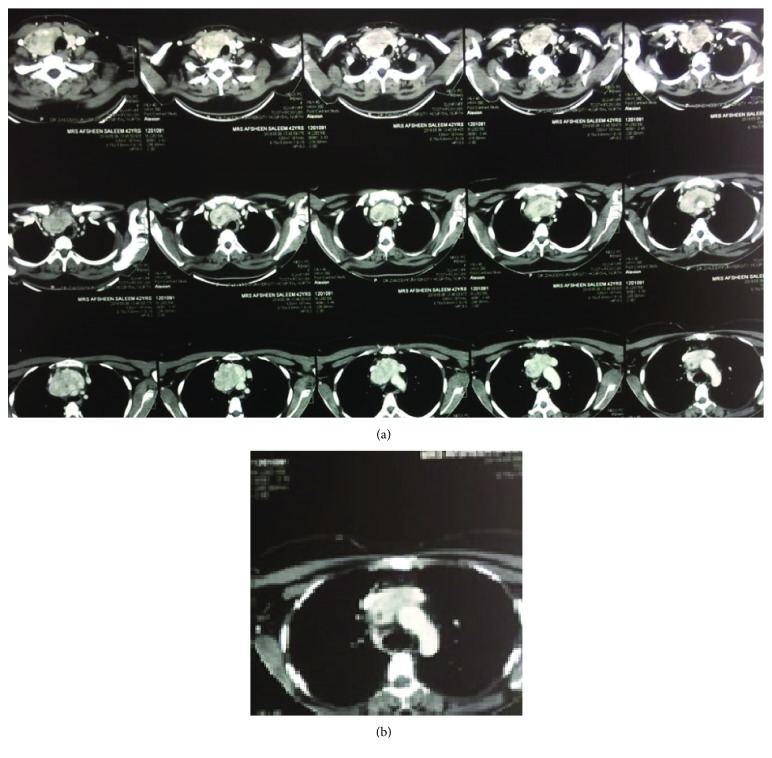
(a) Multiple axial sections of CT scan showing retrosternal extension of thyroid mass. (b) Axial section showing inferior end of mass lying over the arch of aorta.

This 42-year-old female, with no known comorbids, presented to us with complaints of anterior neck swelling, more on the right side which has gradually increased in size over the last 5 years accompanied with shortness of breath especially while climbing stairs which has progressively worsened since the onset of symptoms. She had no complains of dysphonia or dysphagia. On examination, a right anterior neck swelling was present which was firm, approximately 3 × 3 cm in size, nontender, noncompressible, and appears nodular, with overlying skin normal. The rest of the systemic examination was normal. She underwent fine needle aspiration biopsy which showed a benign thyroidal swelling. Computed tomography scan was done which showed large, well circumscribed, multinodular goiter with extension of right lobe and isthmus to superior mediastinum with a size of 8.8 × 6.5 × 4.5 cm (Figure 1).

She was admitted electively and underwent total thyroidectomy with excision of mediastinal component. Initially, thyroid was mobilized with transverse neck incision. Subsequently, the sternotomy was performed and the retrosternal component that was adherent to innominate vein and mediastinal fat was mobilized. The intraoperative findings were enlarged right lobe of thyroid of about 8 × 6 cm and left lobe of about 4 × 3 cm in size. The mass appeared in continuity from neck to mediastinum but separately capsulated sizing to 5 × 5 cm. The postoperative course was unremarkable, and she was discharged on the 3^rd^ postoperative day.

She was found to be doing well up to six-week follow-up, and her histopathology revealed benign nodular hyperplasia of thyroid with adenomatous nodules in the mediastinal thyroid. She was referred to endocrinology service for further management.

## 3. Case 2

**Figure 2:**
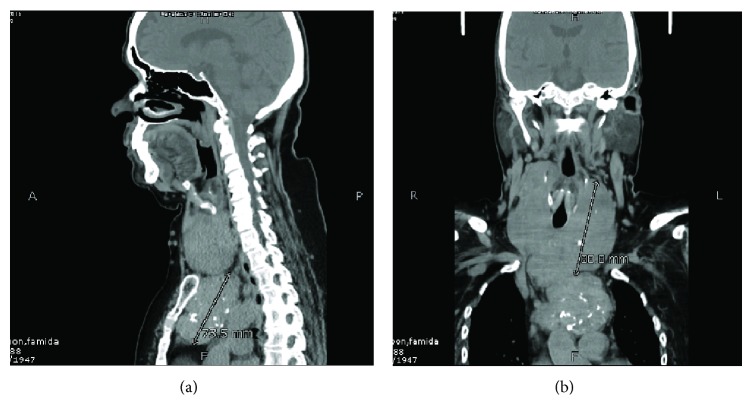
CT scan demonstrating retrosternal extension of thyroid mass in a sagittal and a coronal section.

**Figure 3:**
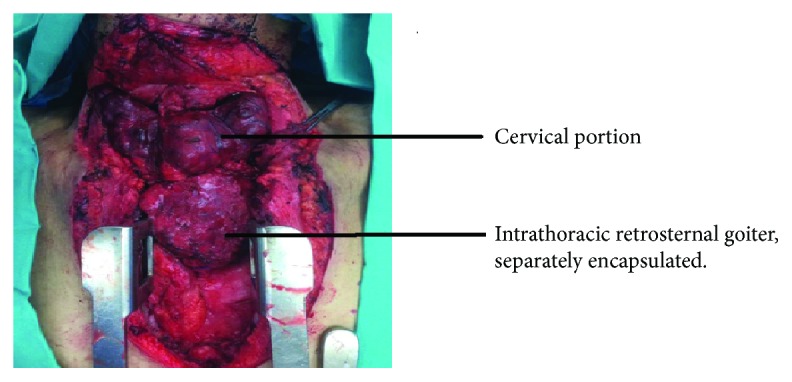
Intraoperative image showing the cervical portion of thyroid mass. Another mass is seen below which is intrathoracic, retrosternal, and separately encapsulated.

This patient is a 68-year-old female, known case of hypertension for the last eight years, presented to us with complaints of anterior neck swelling for about 40 years which had gradually started increasing in size for the last four years. She developed progressive difficulty in swallowing and breathing for the last three months. On examination, there was a presence of large neck swelling, multinodular, which moved on deglutition, with lower limit of swelling not palpable. Prominent dilated veins were appreciated on the neck. A computed tomography scan was done which showed enlarged thyroid with multiple internal calcifications and retrosternal extension up to the level of ascending aorta with multiple collateral vascular channels around mass lesion in anterior mediastinum (Figure 2). She also underwent total thyroidectomy, sternotomy, and excision of mass lesion. The intraoperative findings were enlarged multinodular goiter with thyroid gland reaching the manubrium. The mediastinal component was also large and separately capsulated from cervical component, extending up to the arch of aorta and superior vena cava with compression of brachiocephalic vein (Figure 3). The mass was carefully dissected from the above vessels. Specimen was sent for histopathology. Postoperatively, the patient remained well. She was given intravenous analgesia and deep venous thrombosis prophylaxis. She developed respiratory distress on 2^nd^ post-op day, and a chest X-ray showed elevation of the right hemidiaphragm (most likely due to iatrogenic right phrenic nerve injury) and right lower lobe atelectasis and hence was shifted to the intensive care unit for observation. She was managed conservatively with chest physiotherapy, nebulizers, and application of BIPAP. She responded to supportive therapy and recovered well. She also developed asymptomatic hypocalcaemia and was managed with both intravenous and oral replacement. She was discharged from the hospital on eighth postoperative day.

She did well on follow-ups. She was kept on oral thyroxin and calcium. Her histopathology revealed benign nodular hyperplasia with degenerative changes in both tissues with lymph nodes showing benign reactive changes. Both tissues were negative for malignancy. She was also advised to continue regular follow-ups in endocrinology clinic for further management.

## 4. Discussion

The cervicomediastinal goiters, which are usually the extension of a single lobe of thyroid gland into the mediastinum, usually present with compressive symptoms of upper airway and esophageal tract with rapidly expanding size mimicking a malignant disease [1]. Recent studies reported that patients with cervicomediastinal thyroid masses most commonly present with neck mass and shortness of breath in more than 65% of cases with dysphagia occurring in about 25-30% of cases and thyrotoxic symptoms occur in only about 10-12.5% of patients [1, 2]. Therefore, these thyroid masses with or without respiratory distress require surgical excision as the only method of treatment as there is no other way of halting its progress and preventing it from compression of other mediastinal structures [1, 5]. In both of our patients, there was a complex thyroid lesion with retrosternal component and symptoms due to pressure effect.

Computed tomography (CT) scan remains the standard imaging conducted before operating on the cervicomediastinal thyroid masses to determine the size and extent of the mass and its relation to the surrounding mediastinal structures. It therefore helps in preoperating planning for anesthesia and surgical access either through cervical collar incision or cervicosternotomy for safe and complete removal of the mass [5, 6]. We anticipated that this patient will require sternotomy, and a multidisciplinary approach was used. In literature, a criteria has been defined for selecting patients for sternotomy based on CT features which include the volume of thyroid gland and whether it has extension below the carina, the source of its blood supply and the risk of hemorrhage, and the presence of enlarged mediastinal lymph nodes as in the presence of malignancy [6]. Although 97% of mediastinal goiters can be delivered through cervical approach, it is sometimes evident from imaging to predict the need of sternotomy for the complete and safe resection with recent studies reporting the rate of sternotomies to be about 3-8% [5]. Even with combined cervicosternotomy approach, the prognosis of the patients in our cases as well as in literature has been excellent with almost zero percent mortality resulting in immediate resolution of symptoms [1, 5, 6]. These separately capsulated masses if attempted to pull through cervical incision may possibly result in catastrophic bleeding with grave consequences.

This standard approach described above also eliminates the risk of “forgotten goiter” which is an extremely rare condition, a thyroid mass not directly in connection with cervical goiter, but present in mediastinum, separately encapsulated, as the two cases that we described above. If not removed and missed at the time of initial excision of cervical thyroid may result in reoperation later on, increasing the risk of morbidity and mortality from the procedure, and has been associated with higher risk of complications [2, 6, 7]. Therefore, detailed examination and extensive imaging preoperatively keeping in mind the possibility of a separate retrosternal thyroid mass can result in better preoperative planning and patient counseling, hence further reducing the morbidity and risk of complications from the procedure.

## 5. Conclusion

These patients presenting with a cervicomediastinal masses usually need multidisciplinary team approach in the setting of a tertiary care hospital. These two cases highlight the importance of multidisciplinary approach keeping in mind the possibility of retrosternal extension as a separate entity and attempt to deliver through cervical approach may lead to increased complications.
